# Patterns of Immune Infiltration in Endometriosis and Their Relationship to r-AFS Stages

**DOI:** 10.3389/fgene.2021.631715

**Published:** 2021-06-18

**Authors:** Qiyu Zhong, Fan Yang, Xiaochuan Chen, Jinbo Li, Cailing Zhong, Shuqin Chen

**Affiliations:** ^1^Department of Gynecology, The Sixth Affiliated Hospital of Sun Yat-sen University, Guangzhou, China; ^2^Department of Gynecology and Obstetrics, The First Affiliated Hospital, Sun Yat-sen University, Guangzhou, China

**Keywords:** endometriosis, immune infiltration, GEO, M2 macrophages, r-AFS stages

## Abstract

**Background:** Endometriosis (EMS) is an estrogen-dependent disease in which endometrial glands and stroma arise outside the uterus. Current studies have suggested that the number and function of immune cells are abnormal in the abdominal fluid and ectopic lesion tissues of patients with EMS. The developed CIBERSORT method allows immune cell profiling by the deconvolution of gene expression microarray data.

**Methods:** By applying CIBERSORT, we assessed the relative proportions of immune cells in 68 normal endometrial tissues (NO), 112 eutopic endometrial tissues (EU) and 24 ectopic endometrial tissues (EC). The obtained immune cell profiles provided enumeration and activation status of 22 immune cell subtypes. We obtained associations between the immune cell environment and EMS r-AFS stages. Macrophages were evaluated by immunohistochemistry (IHC) in 60 patients with ovarian endometriomas.

**Results:** Total natural killer (NK) cells were significantly decreased in EC, while plasma cells and resting CD4 memory T cells were increased in EC. Total macrophages in EC were significantly increased compared to those of EU and NO, and M2 macrophages were the primary macrophages in EC. Compared to those of EC from patients with r-AFS stage I ~ II, M2 macrophages in EC from patients with stage III ~ IV were significantly increased. IHC experiments showed that total macrophages were increased in EC, with M2 macrophages being the primary subtype.

**Conclusions:** Our data demonstrate that deconvolution of gene expression data by CIBERSORT provides valuable information about immune cell composition in EMS.

## Introduction

Endometriosis (EMS) is a chronic inflammatory disease defined as the presence of endometrial tissues outside the uterus, that causes pelvic pain and infertility (Chapron et al., 2019; Laganà et al., 2019). EMS affects nearly 10% of women of reproductive age, and causes a significant economic burden, costing $70 billion dollars annually in the United States alone (Simoens et al., 2012; Wang et al., 2020). Although EMS is a benign disease, it exhibits malignant behaviors such as infiltration and growth, implant metastasis and relapse tendency.

In 1979, the American Fertility Society (AFS) proposed a classification system for EMS, which was revised in 1985. The revised American Fertility Society (r-AFS) classification system is the most commonly used clinical classification system for EMS (Zeng et al., 2014). Based on clinical experience and scientific finding, this classification system can describe any case, has an associated paper form to encourage complete documentation, is quantitative to allow for analysis, and has assigned cutoff points, reflecting severity of EMS to a certain extent. The specific classification is as follows: 1~5 were divided into stage I (minor lesions); 6~15 were divided into stage II (mild lesions); 16~40 were stage III (moderate lesion); and >40 were divided into stage IV (severe lesion).

The decrease in quality of life and working days, the increase in surgical intervention and the assisted reproductive technology rate caused by EMS contributes to high social costs (Vitagliano et al., 2016). Therefore, EMS has become an urgent social problem to be solved, but its pathogenesis is still unclear.

Recently, increasing attention has been given to the study of immune factors related to EMS. As early as 1981, Dmowski et al. proposed that the composition of local immune cells affected the occurrence and development of EMS (Dmowski et al., 1981). Some studies suggest that the number and function of immune cells change significantly in the peritoneal fluid and ectopic endometrial tissues of patients with EMS, and these abnormal immune mechanisms may play an important role in the occurrence and development of EMS. It was found that expression levels of some immune genes related to immune cell infiltration, cell adhesion and the interactions between cytokines and their receptors in ectopic lesions were increased (Ahn et al., 2015).

EMS is an immune inflammatory disease, and the abnormal number and function of various immune cells in the abdominal cavity environment improves the invasion and adhesion ability of endometrial cells, including natural killer (NK) cells, macrophages, dendritic cells, mast cells, T cells, etc., promoting implantation of ectopic endometrial cells, angiogenesis and the establishment and maintenance of ectopic lesions, which can cause ectopic endometrium to flow back into the pelvic and abdominal cavity with menstrual blood and escape from immune monitoring (Symons et al., 2018).

Previous studies primarily used immunohistochemistry (IHC) and flow cytometry to analyze the composition of immune cells in tissues. However, these experimental methods all depend on the specific recognition of cell surface markers, and the tissues need to be decomposed during flow cytometry, which may lead to the loss of some cells and the distortion of results. To solve these problems, Newman developed the biological software CIBERSORT in 2015 (Newman et al., 2015), which could calculate the composition of immune cells in tissues based on complex gene expression profiles to verify its reliability in colorectal cancer, lung cancer, gastric cancer, breast cancer and other tumors by flow cytometry (Angelova et al., 2015; Ali et al., 2016). Rohr-Udilova et al. (2018) applied the original CIBERSORT gene signature file LM22 which defined 22 immune cell subtypes and analyzed datasets from human hepatocellular carcinoma (HCC), HCC tumor adjacent tissue and healthy livers. Therefore, it can be widely used in the analysis of gene expression profiles of various diseases and saves costs to a certain extent.

In this study, the composition of immune cells in tissues can be obtained using transcriptome data from EMS, and the relationship between immune cells and stages can be further analyzed. Gene expression data of ectopic endometrium and endometrial tissue samples from 309 patients with or without EMS were analyzed. CIBERSORT was used to evaluate the proportion of 22 immune cell types in these endometrial tissues to quantify the composition of cells involved in the immune response in the endometrium and to analyze their relationship with EMS r-AFS classification.

## Materials and Methods

We used R software (version 3.4.4, https://cran.r-project.org/doc/FAQ/R-FAQ.html#Citing-R) and CIBERSORT (https://cibersort.stanford.edu) (Newman et al., 2015) for all bioinformatics analyses in the study.

CIBERSORT implements a machine learning approach called support vector regression (SVR) that improves deconvolution performance through a combination of feature selection and robust mathematical optimization techniques. Unlike previous methods, SVR performs a feature selection, in which genes from the signature matrix are adaptively selected to deconvolve a given mixture (Newman et al., 2015). CIBERSORT is an analytical tool that accurately quantifies the relative levels of distinct immune cell types within a complex gene expression mixture. To characterize and quantify each immune cell subtype, CIBERSORT uses gene expression signatures consistent with 547 genes (Supplementary Table 1, https://cibersort.stanford.edu).

### Gene Expression Datasets

Here, we applied the original CIBERSORT gene signature file LM22 which defined 22 immune cell subtypes and analyzed datasets from ectopic endometrial tissue (EC) and eutopic endometrial tissue (EU) in patients with EMS and normal endometrial tissues (NO).

All microarray gene expression data were obtained from the public GEO database (https://www.ncbi.nlm.nih.gov/gds/) under the following conditions: “endometriosis, series, expression profiling by array, homo.” June 2019 was used as the deadline for searching.

According to the research purpose and research scope of this project, a total of 7 series chips were included: GSE120103, GSE51981, GSE25628, GSE37837, GSE7846, GSE6364, and GSE7305.

A total of 309 cases of gene transcriptome data were downloaded, including 116 cases of NO, 158 cases of EU EMS patients and 35 cases of EC EMS patients, and the corresponding r-AFS classification information for patients was downloaded.

### Data Processing and Immune Cell Infiltration Analysis

Raw data from Affymetrix Human Genome U133 Plus 2.0 cel files and Affymetrix Human Genome U133A 2.0 cel files were processed by using robust multiarray analysis (RMA) method, normalized according to quantiles method and subsequently log-transformed in RMAExpress software (version 1.0.5, http://rmaexpress.bmbolstad.com/). Raw data from Agilent Technologies text files were processed by using the “normexp” function with an offset of 50 for background adjustment, normalized according to quantiles method and subsequently log-transformed in R software. Heterogeneity and latent variables are widely recognized as primary sources of variability and bias in high-throughput experiments, and the most well-known sources of latent variation in genomic experiments are batch effects when samples are performed on different days, by different people or in different groups. Surrogate variable analysis (SVA, http://www.bioconductor.org/packages/release/bioc/html/sva.html) package is used to identify and remove batch effects and other unwanted sources of variation. Data were further normalized using the combat package in SVA to correct for batch effect. The gene expression of each sample before batch normalization is shown in Supplementary Table 2. Principal component analysis (PCA) was performed on the data before (Supplementary Figure 1A) and after (Supplementary Figure 1B) batch normalization. All samples were analyzed for immune cell profiles by CIBERSORT, and the number of permutations was set to 100. Permutation represents times of the algorithm run using the default signature matrix. Theoretically, the accuracy of the results increases with the increase of running times (Newman et al., 2015). Twenty-two immune cell types, together with CIBERSORT metrics, such as the Pearson correlation coefficient, CIBERSORT *p*-value and root mean squared error (RMSE), were quantified for each sample. The CIBERSORT *p*-value reflects the statistical significance of the deconvolution results across all cell subsets and is useful for filtering out deconvolution with less significant fitting accuracy (https://cibersort.stanford.edu). From all samples analyzed, we selected 68/24/112 NO/EC/EU samples (Supplementary Table 3) that met the requirements of CIBERSORT *p*-value < 0.05. The histogram of 68/24/112 NO/EC/EU samples based on immune cell proportions was drawn by “barplot” function in R software (Supplementary Figure 2).

### R-AFS Subtyping and Inference of Infiltrating Immune Cells

According to the r-AFS classification downloaded from the GEO database, 30 samples with missing staging information were removed, and the remaining 82 EU cases of EMS patients were divided into 20 cases of stage I~II endometrial tissue and 62 cases of stage III~IV endometrial tissue (Table 1). The list of selected samples and corresponding GEO accessions is shown in Table 1. The immune cell profile was calculated for each sample, and the upper quartile (P25), median value and lower quartile (P75) for each tissue type (NO, EC and EU) were calculated. Kruskal-Wallis tests were applied to analyze differences among EC, EU and NO. For macrophages, Pearson correlation coefficients with other immune cell types were calculated using SPSS 24.0 software.

**Table 1:** Datasets of different stage EMS.

**Tissues**	**Datasets used for CIBERSORT analysis**	**GEO accessions**
I~II stage (*n =* 20)	GSM1256672 GSM1256674 GSM1256677 GSM1256682 GSM1256683 GSM1256685 GSM1256686 GSM1256688 GSM1256689 GSM1256691 GSM1256692 GSM1256694 GSM1256698 GSM1256700 GSM1256705 GSM1256706 GSM1256708 GSM1256713 GSM1256715 GSM1256778	GSE51981
III~IV stage (*n =* 62)	GSM150191 GSM150193 GSM150195 GSM150203 GSM150211 GSM150213 GSM150215 GSM150217 GSM150218 GSM150219 GSM1256653 GSM1256655 GSM1256660 GSM1256662 GSM1256664 GSM1256668 GSM1256673 GSM1256675 GSM1256676 GSM1256678 GSM1256679 GSM1256681 GSM1256687 GSM1256695 GSM1256696 GSM1256697 GSM1256699 GSM1256701 GSM1256703 GSM1256704 GSM1256707 GSM1256710 GSM1256711 GSM1256712 GSM1256714 GSM1256716 GSM1256719 GSM1256780 GSM928781 GSM928783 GSM928789 GSM928791 GSM928793 GSM928795 GSM928797 GSM928801 GSM928803 GSM928805 GSM928809 GSM928813 GSM3393501 GSM3393502 GSM3393504 GSM3393505 GSM3393506 GSM3393508 GSM3393520 GSM3393521 GSM3393522 GSM3393524 GSM3393525 GSM3393526	GSE120103 GSE51981 GSE37837 GSE6364

The total macrophage fraction was calculated as the sum of M0, M1, and M2 macrophage fractions. Total T cells were calculated as the sum of CD8 T cells, naive CD4 T cells, resting CD4 memory T cells, activated CD4 memory T cells, follicular helper T cells, regulatory T cells (Tregs) and gamma delta T cells.

### Patients and Specimens

A total of 60 anonymized patients with ovarian endometriomas and 30 anonymized patients without EMS were enrolled and pathologically confirmed by the Pathology Department of the First Affiliated Hospital of Sun Yat-sen University. All patients with EMS of childbearing age without hormone or drug treatment in the last 6 months underwent laparoscopic ovarian cyst removal from December 2017 to December 2019. Ectopic endometrial tissue and eutopic endometrial tissue were collected from the same patient with ovarian endometriomas and classified according to the r-AFS system. Paraffin sections of endometrial tissues as the control group were taken from patients without EMS who underwent laparoscopic myomectomy. Normal endometrial tissue and eutopic endometrial tissue were obtained by endometrial biopsy. Ectopic endometrial tissue was obtained by laparoscopic ovarian endometriomas resection. The pathology of endometrial tissue specimens was proliferative endometrium. Endometrial hyperplasia or malignant transformation, previous history of EMS surgery or amenorrhea, acute inflammation and autoimmune disease were excluded from the study. All specimens were obtained with the informed consent of patients, and this study was approved by the Medical Research Ethics Committee of the First Affiliated Hospital of Sun Yat-sen University. Clinical data were obtained in a well-designed questionnaire, including age, body mass index (BMI), menstrual cycle length in days, number of days of menstrual bleeding, dysmenorrhea visual analog scale (VAS) score and r-AFS score (**Tables 4**, **5**). The VAS consists of a line usually 100 mm in length, with anchor descriptors such as “no pain” and “worst pain imaginable.” BMI is a statistical index using a person's weight and height to provide an estimate of body fat. It is calculated by taking a person's weight, in kilograms, divided by their height, in meters squared, or BMI = weight (in kg)/ height^2^ (in m^2^).

### IHC

To explore the distribution of macrophages in eutopic and ectopic endometrium of patients with EMS, immunohistochemical experiments were conducted to determine the integral optic density (IOD) values of CD68 and CD206 in endometrial tissues of the control group and eutopic and ectopic endometrium of patients with EMS to calculate the ratio of CD206/CD68.

Total macrophages and M2 macrophages were evaluated immunohistochemically using staining for CD68 and CD206, respectively. All endometrial tissues were fixed with 10% neutral buffered formalin fixative, dehydrated and embedded in paraffin. Wax blocks were sectioned into 4 μm continuous sections, and slides were randomly selected for immunohistochemical staining.

For IHC, 4-μm paraffin sections were baked at 60°C for 1 h, deparaffinized in xylene and rehydrated via graded ethanol. Then slides were microwaved in citric buffer (10 mM citric acid, pH 6.0) and cooled at room temperature to retrieve antigens. Endogenous peroxidase activity was blocked by 3% H_2_O_2_, and non-specific binding sites were blocked by 10% goat serum.

The following primary antibodies were used: mouse anti-CD68 antibody (ab31630; Abcam, USA; 1:200 dilution, cytoplasmic or/and membrane staining) and rabbit anti-CD206 antibody (ab64693; Abcam, USA; 1:1,000 dilution, cytoplasmic or/and membrane staining). Next, the primary antibody against CD68 or CD206 were added to the specimen sections at 4°C and incubated overnight. The primary antibody was removed, and the secondary antibody was incubated with the samples (1:1,000) at 37°C for 30 min. The immunohistochemical MaxVision kit solution was incubated with tissue samples at room temperature for 20 min, and thereafter, 3, 3′-diaminobenzidine (DAB) chromogenic reagent was added. Finally, hematoxylin staining was performed. The primary antibody was replaced with phosphate buffered saline, which was used as the negative control. IHC staining of positive samples was repeated twice.

The results of slides were identified by a double-blind method, and immunohistochemical staining results of all slides were independently judged by two senior pathologists. Using semi-quantitative integration method, six visual fields were randomly selected under a high-power microscope, and immunohistochemical results are expressed by the average IOD of six random visual fields: IOD = density (mean) ^*^ area. Density reflects the concentration or intensity of positive protein, and IOD reflects the total protein expression in the selected area. For measurement IOD, the image system comprised a Leica CCD camera DFC420 connected to a Leica DM IRE2 microscope (Leica Microsystems Imaging Solutions Ltd, Cambridge, United Kingdom). Photographs of representative fields were captured under high-power magnification (×400) using Leica QWin Plus v3 software. The IODs of in each image were measured using Image-Pro Plus 6.0 software (Media Cybernetics Inc., Bethesda, MD, USA).

### Statistical Analysis

The median value (P25-P75) was used to express the fraction of three groups of different immune cells obtained from the GEO database, and the Kruskal-Wallis test was used to compare them because the data didn't conform to normal distribution and homogeneity of variance. The pearson correlation test or spearman rank correlation test was used to analyze the correlation between the two groups of quantitative data. SPSS 24.0 statistical software was used for data analysis. Counting data were expressed by percentage. Qualitative data were described by frequency, and measurement data were measured by nonparametric mean test (X^2^ test and *t*-test). Results were considered statically significant when *p* < 0.05.

## Results

**Figure 1:**
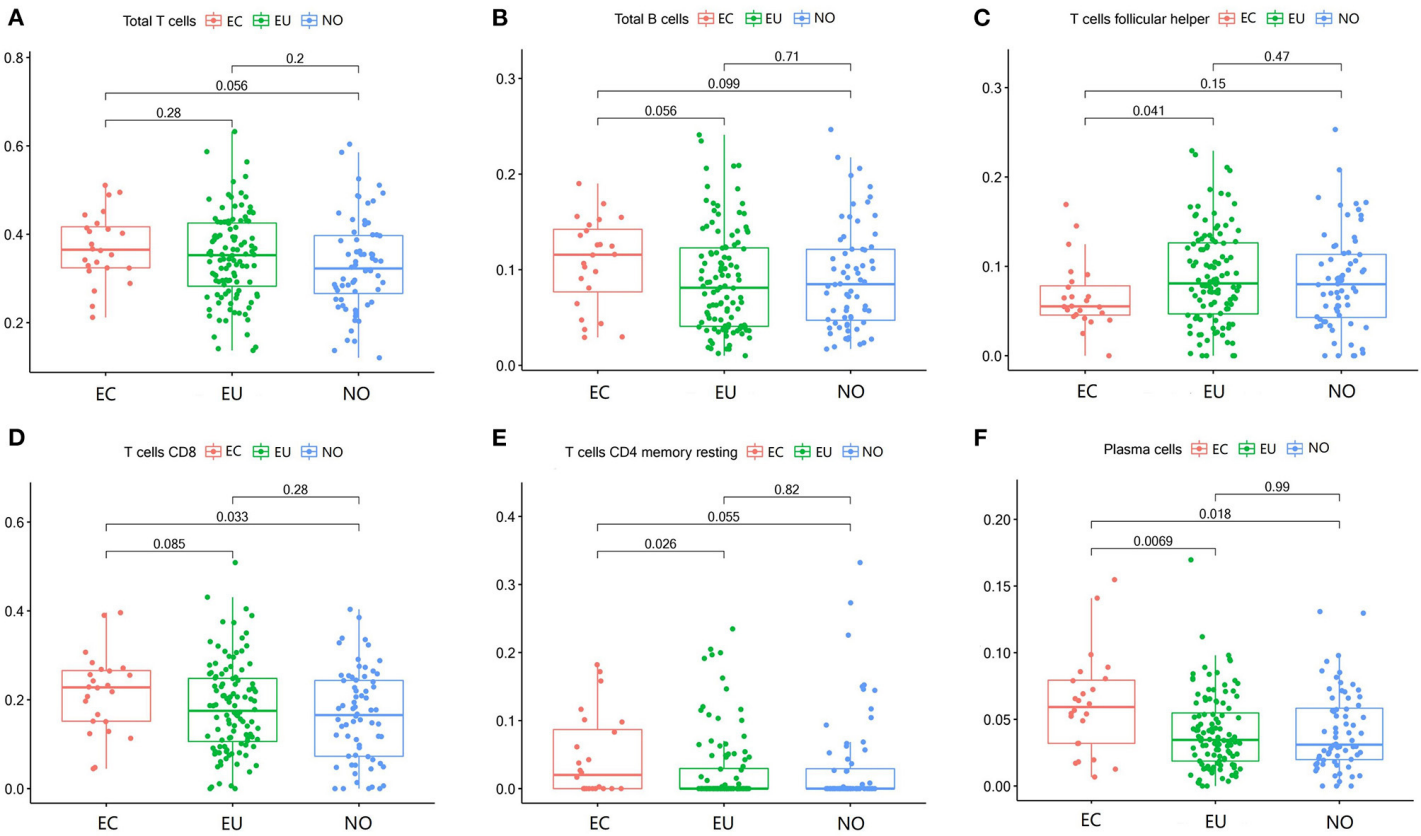
T cells and B cells in EC, EU, and NO. CIBERSORT immune cell fractions were determined for each patient; each dot represents one patient. The upper quartile (P25), median value, lower quartile (P75) after removing datas with large deviation for each cell subset, including total T cells **(A)**, total B cells **(B)**, and follicular helper T cells **(C)**, CD8 T cells **(D)**, resting CD4 memory T cells **(E)**, and plasma cells **(F)** were calculated for each patient group and compared using the Kruskal-Wallis test. Y axes represent the composition ratio of each immune cell.

**Figure 2:**
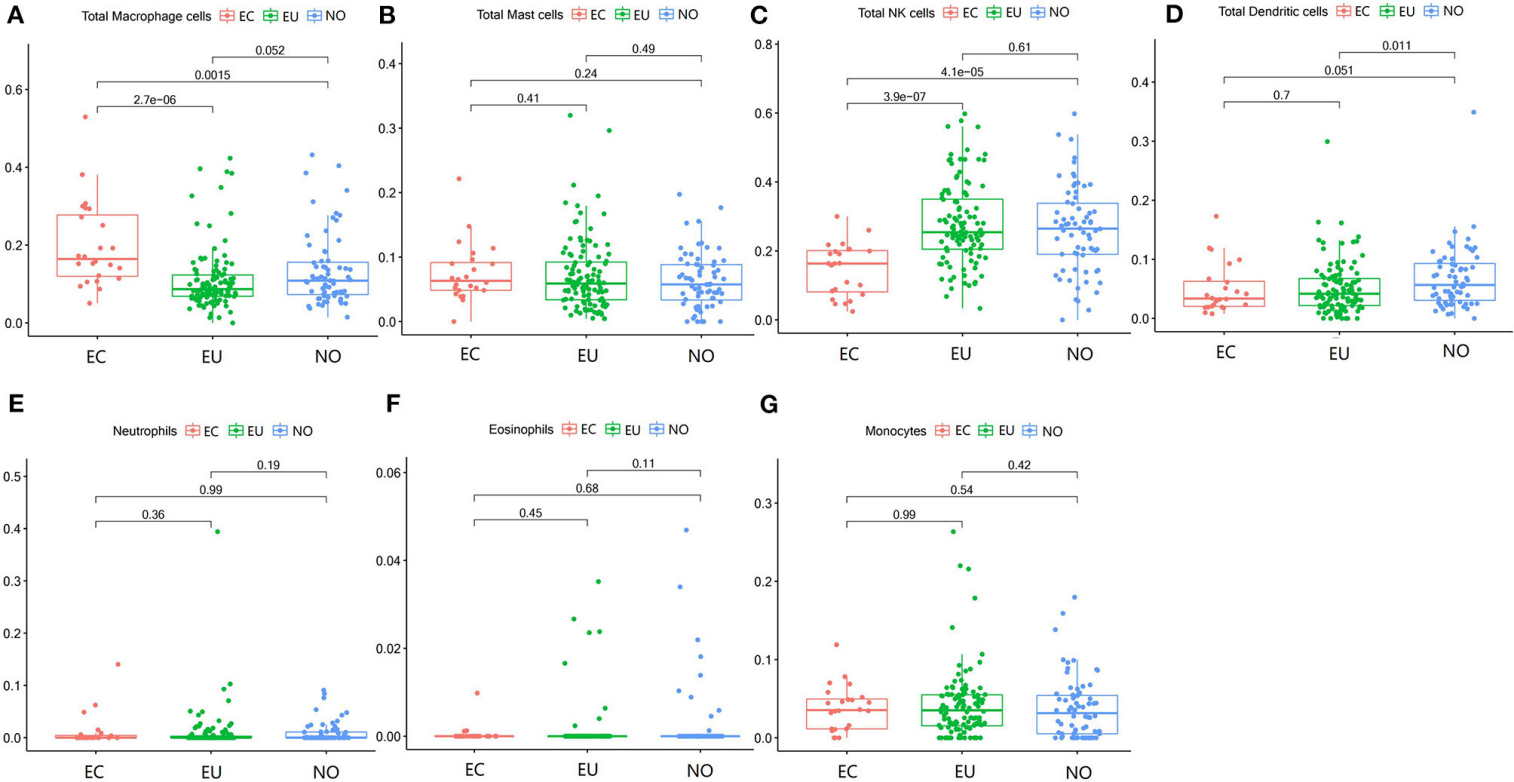
Innate immune cells in EC, EU, and NO. CIBERSORT immune cell fractions were determined for each patient; each dot represents one patient. The upper quartile (P25), median value, lower quartile (P75) after removing datas with large deviation for each cell subset including total macrophages **(A)**, mast cells **(B)**, NK cells **(C)**, dendritic cells **(D)**, neutrophils **(E)**, eosinophils **(F)**, and monocytes **(G)** were calculated for each patient group and compared using the Kruskal-Wallis test. Y axes represent the composition ratio of each immune cell.

**Figure 3:**
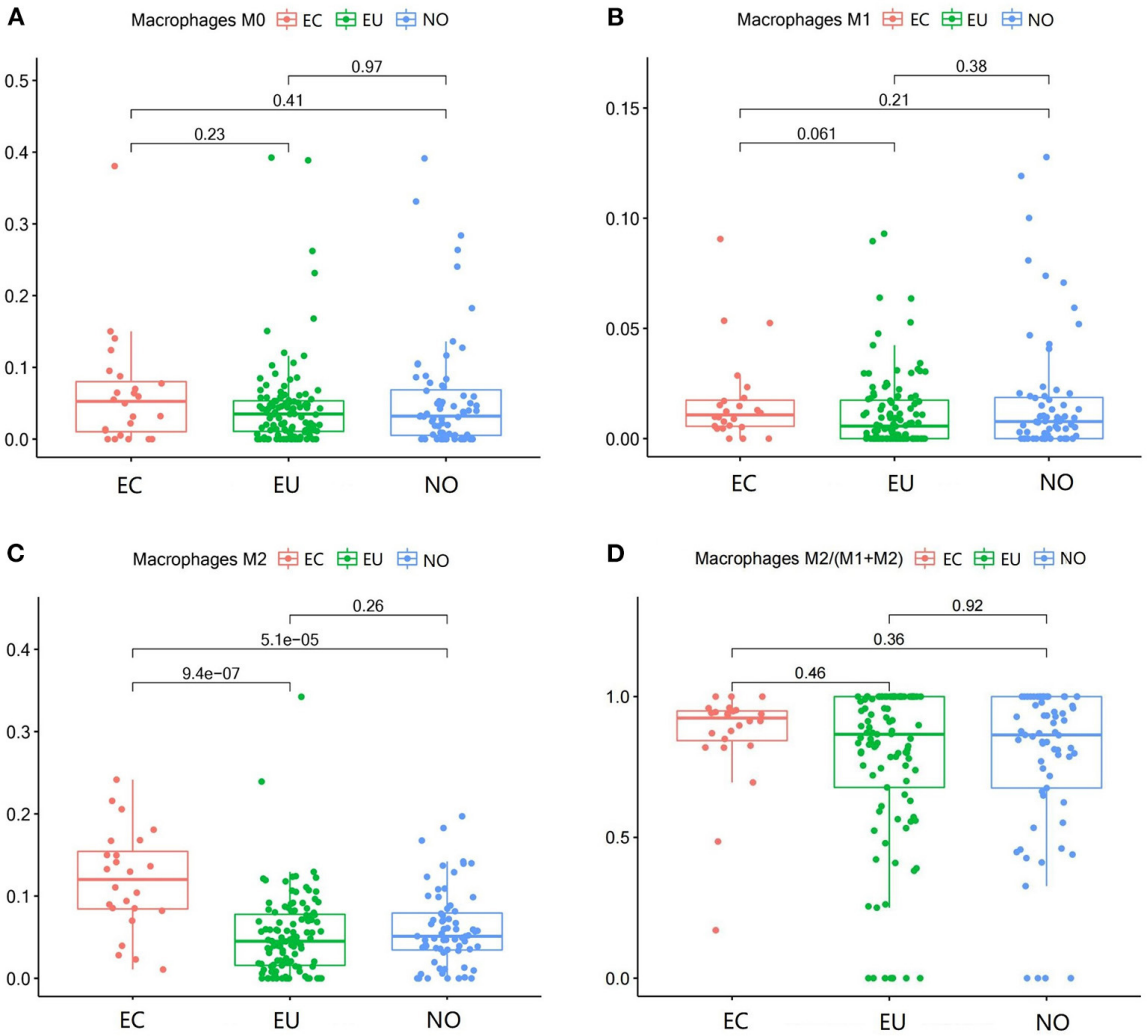
Macrophage subgroups in EC, EU, and NO. CIBERSORT immune cell fractions were determined for each patient; each dot represents one patient. The upper quartile (P25), median value, lower quartile (P75) after removing datas with large deviation for each cell subset, including M0 macrophages **(A)**, M1 macrophages **(B)**, M2 macrophages **(C)**, and M2 macrophages/ (M1 macrophages + M2 macrophages) **(D)**, were calculated for each patient group and compared using the Kruskal-Wallis test. Y axes represent the composition ratio of each immune cell.

**Figure 4:**
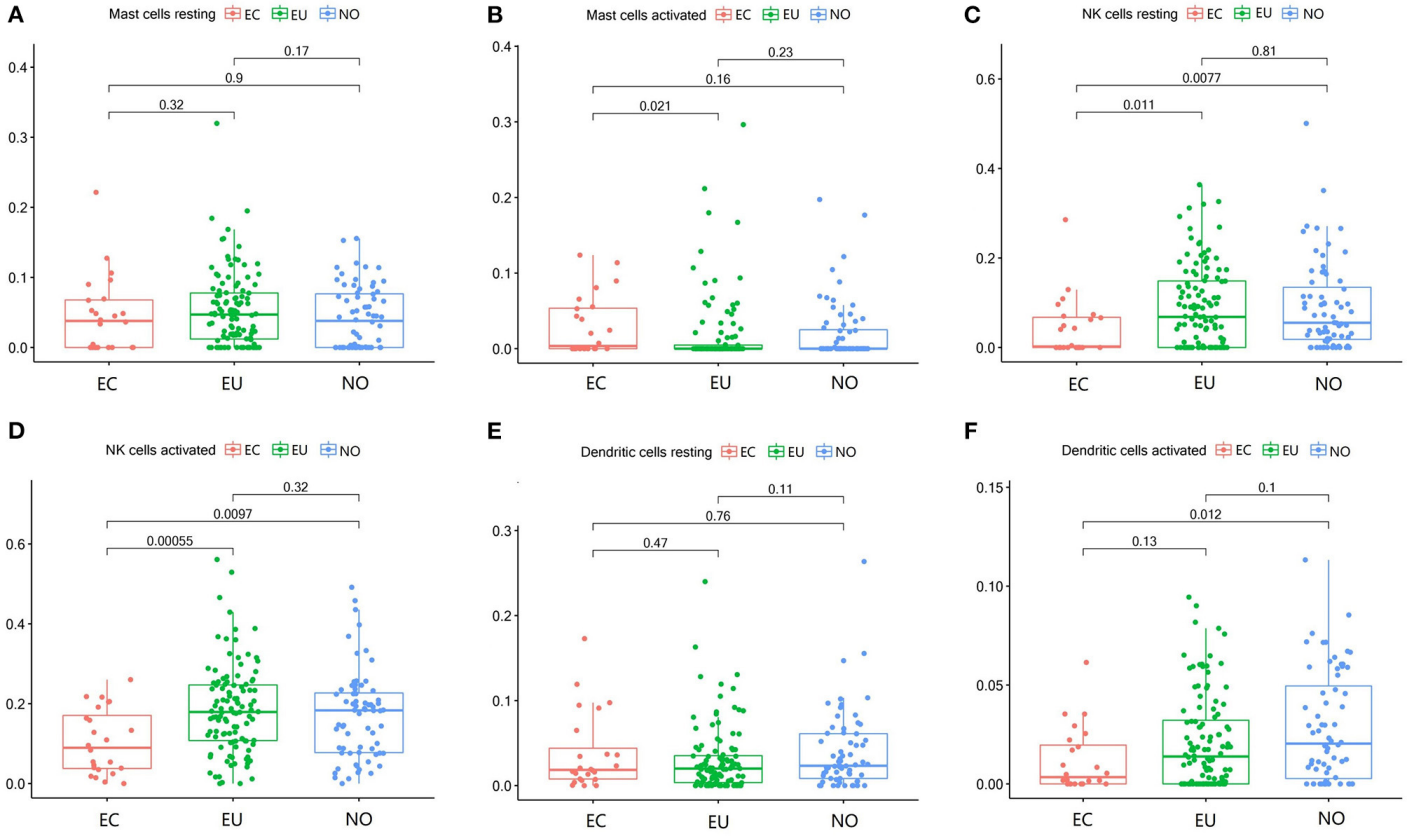
Mast cell, dendritic cells and NK cell subgroups in EC, EU, and NO. CIBERSORT immune cell fractions were determined for each patient; each dot represents one patient. The upper quartile (P25), median value, lower quartile (P75) after removing datas with large deviation for each cell subset including resting mast cells **(A)**, activated mast cells **(B)**, resting NK cells **(C)**, activated NK cells **(D)**, resting dendritic cells **(E)**, and activated dendritic cells **(F)** were calculated for each patient group and compared using the Kruskal-Wallis test. Y axes represent the composition ratio of each immune cell.

A total of 309 cases of gene transcriptome data were downloaded from the GEO database, including 116 cases of NO, 158 cases of EU and 35 cases of EC of EMS patients. After screening at *p* < 0.05 of CIBERSORT analysis, 105 samples were eliminated, and 68 cases of NO, 24 cases of EC and 112 cases of EU remained (Supplementary Table 3). The scatter diagram (Figures 1–**4**) in the three groups and correlation diagram of the proportion of each immune cell type in EC are shown below (**Figure 6**).

**Figure 5:**
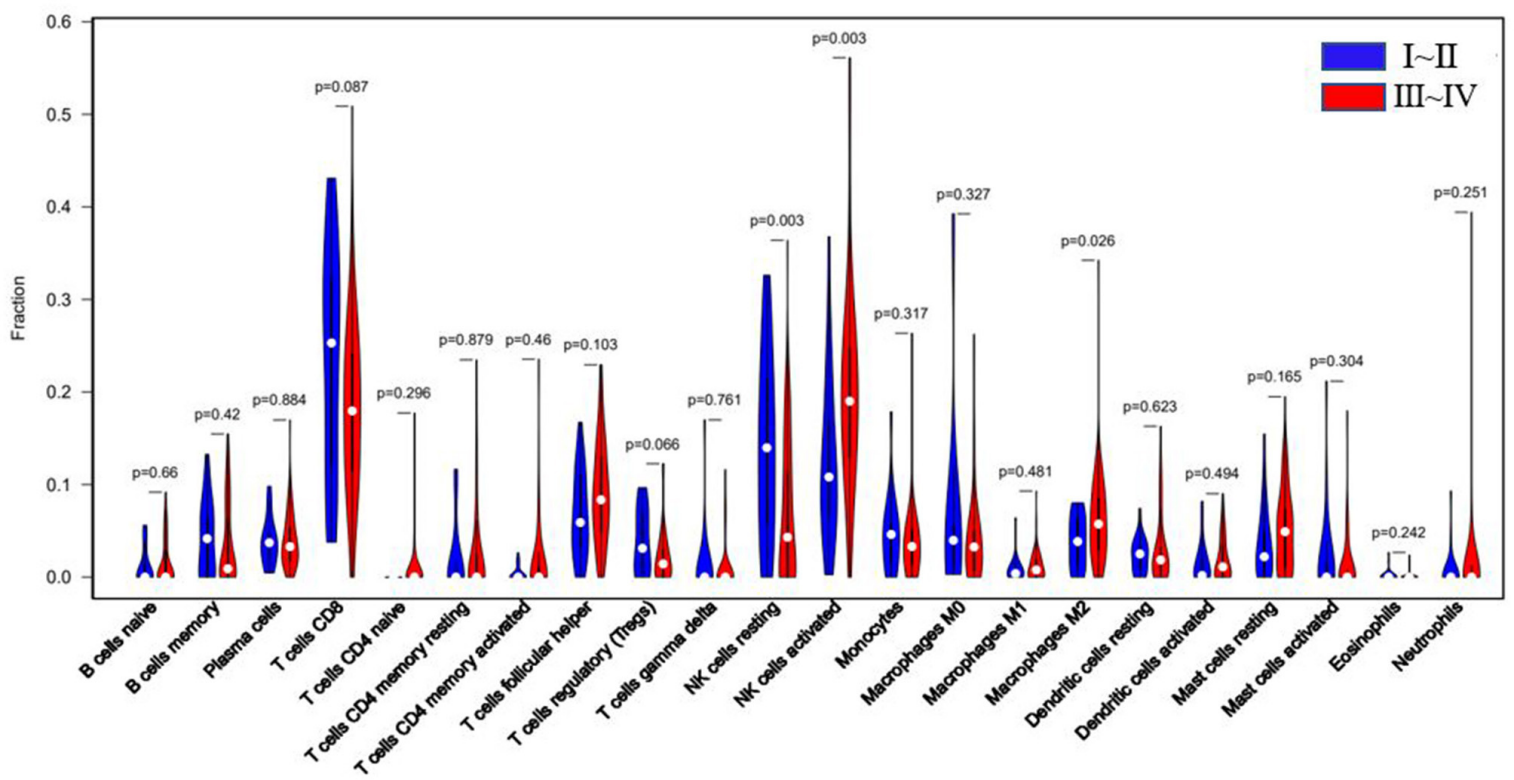
Blue: EU tissue of EMS stage I~II; red: EU tissue of EMS stage III~IV. White dots represent median values of the immune cell fraction.

According to r-AFS classification and staging information downloaded from the GEO database, 30 samples with missing information of r-AFS classification were removed, and the remaining 82 EMS patients were divided into 20 cases of stage I~II and 62 cases of stage III~IV endometrial tissues (Table 1). A violin diagram of the proportion of each immune cell in the samples is shown in **Figure 5**.

### T Cells and B Cells in EC

The fraction of follicular helper T cells was lower in EC than in EU (*p* = 0.041) (Figure 1C, Table 2). The fractions of CD8 T cells, resting CD4 memory T cells and plasma cells in EC were higher than that in EU and NO (Figures 1D–F, Table 2). There was no significant difference in other T cell subsets and total T cells among the three groups (Figure 1A, Table 2). There was no significant difference in the fractions of total B cells (Figure 1B, Table 2) and B memory cells among the three tissues (Table 2). The fraction of naive B cells in all three tissues was low (Table 2).

**Table 2:** Comparison of CIBERSORT immune cell fractions between ectopic endometrial tissue (EC), eutopic endometrial tissue (EU), and normal endometrial tissues (NO).

**Immune cell type**	**CIBERSORT fractions of all infiltrating immune cells**					
	**Median** **±** **Inter-quartile range**			***p*****-values (with Kruskal-Wallis)**		
	**NO**	**EU**	**EC**	**NO vs. EU**	**NO vs. EC**	**EU vs. EC**
Total T cells	0.323 ± 0.141	0.353 ± 0.151	0.365 ± 0.098	0.2	0.056	0.28
T cells CD8	0.166 ± 0.172	0.175 ± 0.146	0.228 ± 0.116	0.28	0.033	0.085
T cells CD4 memory resting	0.000 ± 0.034	0.000 ± 0.032	0.020 ± 0.094	0.82	0.055	0.026
T cells CD4 memory activated	0.000 ± 0.000	0.000 ± 0.005	0.000 ± 0.000	0.48	0.78	0.42
T cells follicular helper	0.080 ± 0.103	0.081 ± 0.081	0.055 ± 0.037	0.47	0.15	0.041
Tregs	0.009 ± 0.035	0.014 ± 0.036	0.022 ± 0.036	0.88	0.6	0.6
T cells gamma delta	0.000 ± 0.000	0.000 ± 0.000	0.000 ± 0.000	0.21	0.19	0.53
T cells CD4 naive	0.000 ± 0.000	0.000 ± 0.000	0.000 ± 0.000	0.57	0.6	0.39
Total B cells	0.085 ± 0.076	0.081 ± 0.082	0.116 ± 0.077	0.71	0.099	0.056
B cells memory	0.004 ± 0.043	0.010 ± 0.068	0.027 ± 0.071	0.35	0.27	0.61
B cells naive	0.004 ± 0.044	0.000 ± 0.019	0.000 ± 0.016	0.056	0.19	0.99
Plasma cells	0.031 ± 0.040	0.035 ± 0.038	0.059 ± 0.048	0.99	0.018	0.0069
Total Macrophages	0.109 ± 0.087	0.087 ± 0.057	0.164 ± 0.172	0.052	0.0015	2.7e^−6^
M0 macrophages	0.032 ± 0.067	0.035 ± 0.044	0.053 ± 0.078	0.97	0.41	0.23
M1 macrophages	0.008 ± 0.019	0.006 ± 0.018	0.011 ± 0.013	0.38	0.21	0.061
M2 macrophages	0.051 ± 0.047	0.045 ± 0.064	0.120 ± 0.080	0.26	5.1e^−5^	9.4e^−7^
M2/(M1+M2)macrophages	0.864 ± 0.331	0.866 ± 0.335	0.924 ± 0.119	0.92	0.36	0.46
Total Mast cells	0.058 ± 0.057	0.059 ± 0.060	0.063 ± 0.046	0.49	0.24	0.41
Mast cells resting	0.038 ± 0.077	0.047 ± 0.067	0.038 ± 0.069	0.17	0.9	0.32
Mast cells activated	0.000 ± 0.027	0.000 ± 0.005	0.004 ± 0.055	0.23	0.16	0.021
Neutrophils	0.000 ± 0.011	0.000 ± 0.003	0.000 ± 0.005	0.19	0.99	0.36
Total dendritic cells	0.057 ± 0.066	0.042 ± 0.047	0.034 ± 0.046	0.011	0.051	0.7
Dendritic cells resting	0.023 ± 0.053	0.020 ± 0.032	0.018 ± 0.051	0.11	0.76	0.47
Dendritic cells activated	0.020 ± 0.051	0.014 ± 0.033	0.003 ± 0.021	0.1	0.012	0.13
Monocytes	0.032 ± 0.049	0.035 ± 0.041	0.035 ± 0.040	0.42	0.54	0.99
Eosinophils	0.000 ± 0.000	0.000 ± 0.000	0.000 ± 0.000	0.11	0.68	0.45
Total NK cells	0.265 ± 0.152	0.254 ± 0.150	0.163 ± 0.127	0.61	4.1e^−5^	3.9e^−7^
NK cells resting	0.055 ± 0.126	0.068 ± 0.149	0.002 ± 0.069	0.81	0.0077	0.011
NK cells activated	0.183 ± 0.154	0.179 ± 0.141	0.090 ± 0.150	0.32	0.0097	0.00055

### Innate Immune Cells in EC

The fraction of total macrophages was significantly higher in EC than in EU and NO (*p* = 2.7e^−6^, *p* = 0.0015), but there was no significant difference between NO and EU (*p* = 0.052) (Figure 2A). Compared to EU and EC, total dendritic cells were increased in NO (*p* = 0.011 and *p* = 0.051, respectively), but there was no significant difference between EC and EU (*p* = 0.7) (Figure 2D). In contrast, total NK cells were significantly decreased in EC (*p* = 3.9e^−7^, *p* = 4.1e^−5^) (Figure 2C). The fractions of total mast cells, neutrophils, eosinophils and monocytes were not significantly altered among the tissue types (Figures 2B,E–G).

### Macrophage Subgroups in EC

M2 macrophages were increased in EC compared to NO and EU (*p* = 5.1e^−5^ and *p* = 9.4e^−7^, respectively) (Figure 3C). M2 macrophages/ (M1 macrophages+M2 macrophages) in EC were higher than that in NO or EU (*p* = 0.36 and *p* = 0.46, respectively) (Figure 3D), but there was no significance difference, indicating that M2 macrophage polarization existed in EC. In contrast, neither M0 macrophages nor M1 macrophages were significantly different among tissues (Figures 3A,B).

### Mast Cell, Dendritic Cells, and NK Cell Subgroups in EC

The fraction of activated mast cells in endometrial tissues was low (Figure 4B). Resting NK cells were significantly decreased in EC (*p* = 0.0077, *p* = 0.011), but there was no significant difference between EU and NO tissues (*p* = 0.81), and activated NK cells were also significantly decreased in the EC group (*p* = 0.0097, *p* = 0.00055) (Figures 4C,D). Activated dendritic cells were decreased in EC compared to NO and EU (*p* = 0.012 and *p* = 0.13, respectively) (Figure 3F). The fractions of resting mast cells and resting dendritic cells were not significantly altered among the tissue types (Figures 3A,E).

### Immune Cell Patterns in EMS r-AFS Classification

The r-AFS classification is used to predict the recurrence potential of EMS after surgery, reflecting severity of EMS to a certain extent. Generally speaking, stage III ~ IV exhibits early recurrence and poor prognosis. Therefore, we investigated differences in immune cell patterns of eutopic endometrium between r-AFS stage I ~ II and stage III ~ IV. Fraction of resting NK cells (*p* = 0.003) were decreased in eutopic endometrium from people with stage III ~ IV compared to stage I ~ II (Figure 5, Table 3). In contrast, fractions of activated NK cells (*p* = 0.003) and M2 macrophages (*p* = 0.026) were increased in eutopic endometrium from people with stage III ~ IV (Figure 5, Table 3). M2 macrophages/ (M1 macrophages+M2 macrophages) were higher in eutopic endometrium from people with stage III ~ IV compared to stage I ~ II (*p* = 0.414) (Table 3), but there was no significance difference. The primary immune cells in tissues are follicular helper T cells, NK cells, M2 macrophages and resting mast cells (Figure 5, Table 3). Thus, different EMS r-AFS classifications were associated with distinct immune phenotypes.

**Table 3:** Comparison of immune cell fractions between two eutopic endometrial tissue (EU) subclasses.

**Immune cell type**	**CIBERSORT fractions of all infiltrating immune cells**		
	**Median** **±** **Inter-quartile range**		***p*-values**
	**Subclass I **~** II (*n =* 20)**	**Subclass III **~** IV (*n =* 62)**	**(with Kruskal-Wallis)**
T cells CD8	0.253 ± 0.209	0.180 ± 0.133	0.087
T cells CD4 memory resting	0.000 ± 0.021	0.000 ± 0.030	0.879
T cells CD4 memory activated	0.000 ± 0.001	0.000 ± 0.011	0.46
T cells Follicular Helper	0.059 ± 0.080	0.084 ± 0.085	0.103
Tregs	0.031 ± 0.071	0.014 ± 0.031	0.066
T cells gamma delta	0.000 ± 0.000	0.000 ± 0.000	0.761
T cells CD4 naive	0.000 ± 0.000	0.000 ± 0.000	0.296
B cells memory	0.042 ± 0.067	0.009 ± 0.068	0.42
B cells naïve	0.000 ± 0.024	0.000 ± 0.032	0.66
Plasma cells	0.037 ± 0.038	0.033 ± 0.037	0.884
M0 macrophages	0.040 ± 0.045	0.033 ± 0.039	0.327
M1 macrophages	0.004 ± 0.010	0.008 ± 0.019	0.418
M2 macrophages	0.038 ± 0.058	0.057 ± 0.059	0.026
M2/(M1+M2) macrophages	0.854 ± 0.164	0.895 ± 0.232	0.414
Mast cells resting	0.022 ± 0.053	0.049 ± 0.068	0.165
Mast cells activated	0.000 ± 0.016	0.000 ± 0.005	0.304
Neutrophils	0.000 ± 0.000	0.000 ± 0.005	0.251
Dendritic cells resting	0.025 ± 0.028	0.019 ± 0.029	0.623
Dendritic cells activated	0.002 ± 0.020	0.011 ± 0.037	0.494
Monocytes	0.046 ± 0.047	0.033 ± 0.039	0.317
Eosinophils	0.000 ± 0.000	0.000 ± 0.000	0.24
NK cells resting	0.140 ± 0.179	0.043 ± 0.120	0.003
NK cells activated	0.108 ± 0.124	0.190 ± 0.121	0.003

### Correlation of Immune Cells in EC

**Figure 6:**
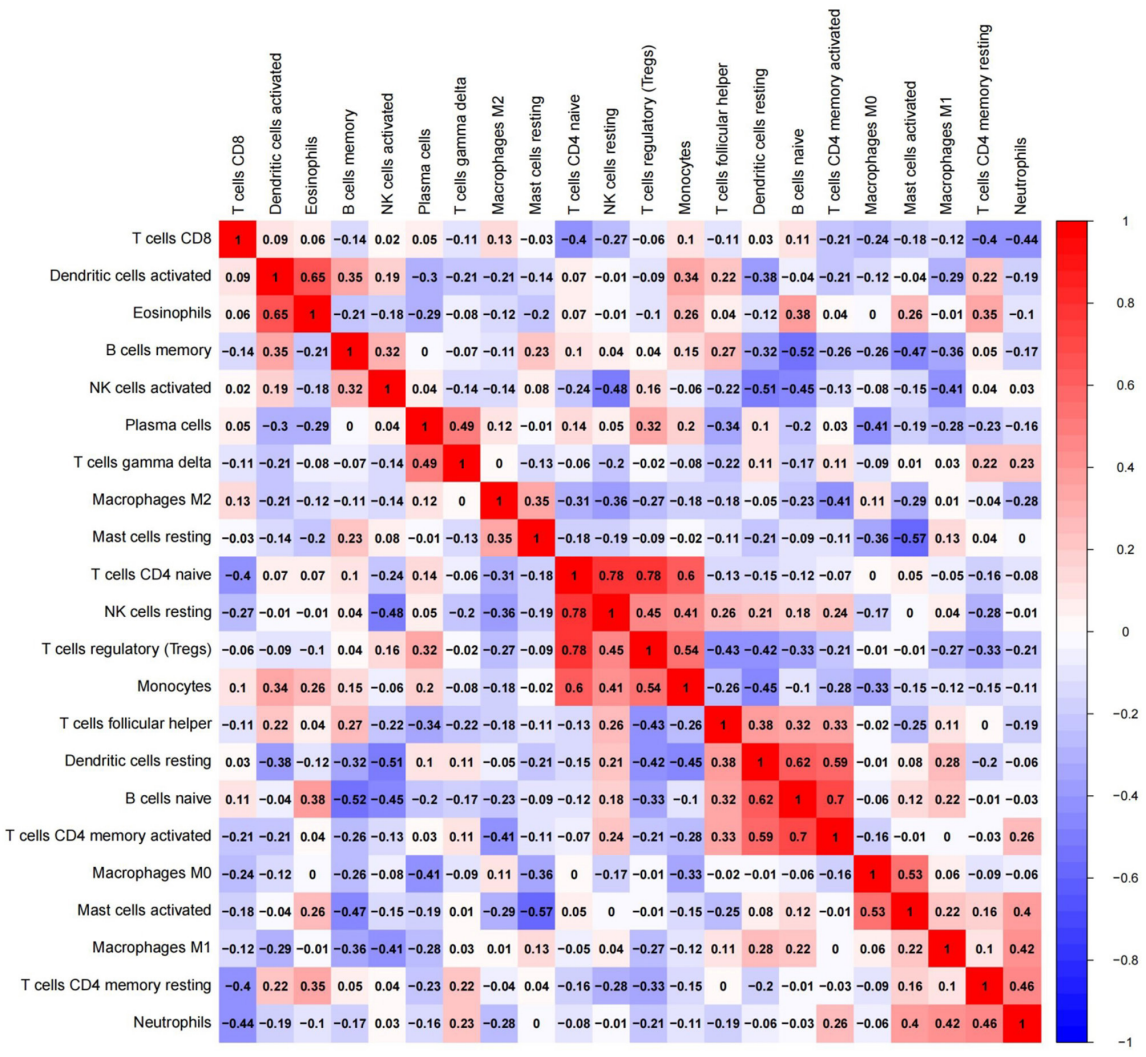
Red: there might be a positive correlation between the two immune cells; blue: there might be a negative correlation between the two immune cells.

To further elucidate immue cell network in EC, we analyzed correlations of different immune cell populations by calculating r^2^ Pearson correlation coefficients (Figure 6). Immune cells with larger correlation coefficients in EC included naive B cells and memory B cells (−0.52); naive B cells and CD4 memory activated T cells (0.7); naive B cells and resting dendritic cells (0.62); resting dendritic cells and CD4 memory activated T cells (0.59); resting dendritic cells and activated NK cells (−0.51); activated mast cells and resting mast cells (−0.57); activated mast cells and M0 macrophages (0.53); naive CD4 T cells and resting NK cells (0.78); naive CD4 T cells and monocytes (0.6); naive CD4 T cells and Tregs (0.78); monocytes and Tregs (0.54); activated dendritic cells and eosinophils (0.65) (Figure 6). Naive B cells correlated positively with CD4 memory activated T cells and resting dendritic cells in EC. However, they correlated negatively with memory B cells in EC. Furthermore, resting dendritic cells correlated positively with CD4 memory activated T cells, and correlated negatively with activated NK cells in EC. Naive CD4 T cells correlated positively with monocytes, resting NK cells and Tregs in EC, and monocytes correlated positively with Tregs. Additionally, activated mast cells correlated positively with M0 macrophages, and correlated negatively with resting mast cells in EC. Activated dendritic cells correlated positively with eosinophils.

### IHC Analysis of Clinical Patient Samples

To verify the exploratory data obtained for macrophages, we conducted immunohistochemical experiments to detect the IOD values of CD68 and CD206 in endometrial tissues of control, eutopic and ectopic endometrium of patients with ovarian endometriomas and calculated the ratio of CD206/CD68, using SPSS 24.0 statistical software to analyze the data.

### Comparison of General Data Among Clinical Patients

Patients with ovarian endometriomas were divided into two groups according to r-AFS stage, including 30 cases of stage I ~ II and 30 cases of stage III ~ IV. Patients in the control group were 24–49 years old with an average age of 36.20 ± 5.40. Patients in stage I ~ II were 24–46 years old with an average age of 32.37 ± 6.19, and patients in stage III ~ IV were 24–48 years old with an average age of 33.93 ± 7.13 (*p* = 0.065). These differences were not statistically significant (Table 4).

**Table 4:** Comparison of general data of clinical patients.

**Group**	***n***	**Age**	**BMI (kg/m^**2**^)**	**Menstrual cycle (d)**	**Menstrual bleeding (d)**
Control	30	36.20 ± 5.40	20.69 ± 1.83	31.40 ± 3.82	5.8 ± 1.75
I ~ II	30	32.37 ± 6.19	20.27 ± 1.72	30.27 ± 2.46	6.37 ± 1.35
III ~ IV	30	33.93 ± 7.13	20.25 ± 1.70	29.87 ± 2.84	6.23 ± 1.28
F		2.823	0.604	1.983	1.211
*p*		0.065	0.549	0.144	0.303

Other basic data, including BMI, menstrual cycle length in days, number of days of menstrual bleeding, etc., showed no significant difference among the three groups (*p* > 0.05) (Table 4). There was no significant difference in VAS scores of dysmenorrhea between stage I ~ II and stage III ~ IV EMS patients (Table 5). The r-AFS scores of patients with stage I ~ II EMS were 2~15, with an average score of 10.03 ± 4.11, while those of patients with stage III ~ IV EMS were 30~132, with an average score of 78.27 ± 26.73 (*p* < 0.001) (Table 5).

**Table 5:** Comparison of VAS score of dysmenorrhea and r-AFS score.

**Group**	**VAS score of dysmenorrhea**	**r-AFS score**
I ~ II	2.33 ± 2.23	10.03 ± 4.11
III ~ IV	2.60 ± 2.57	78.27 ± 26.73
*t*	−0.43	−13.82
*p*	0.669	0.000

### Macrophages in EC of Patients With Ovarian Endometriomas

**Figure 7:**
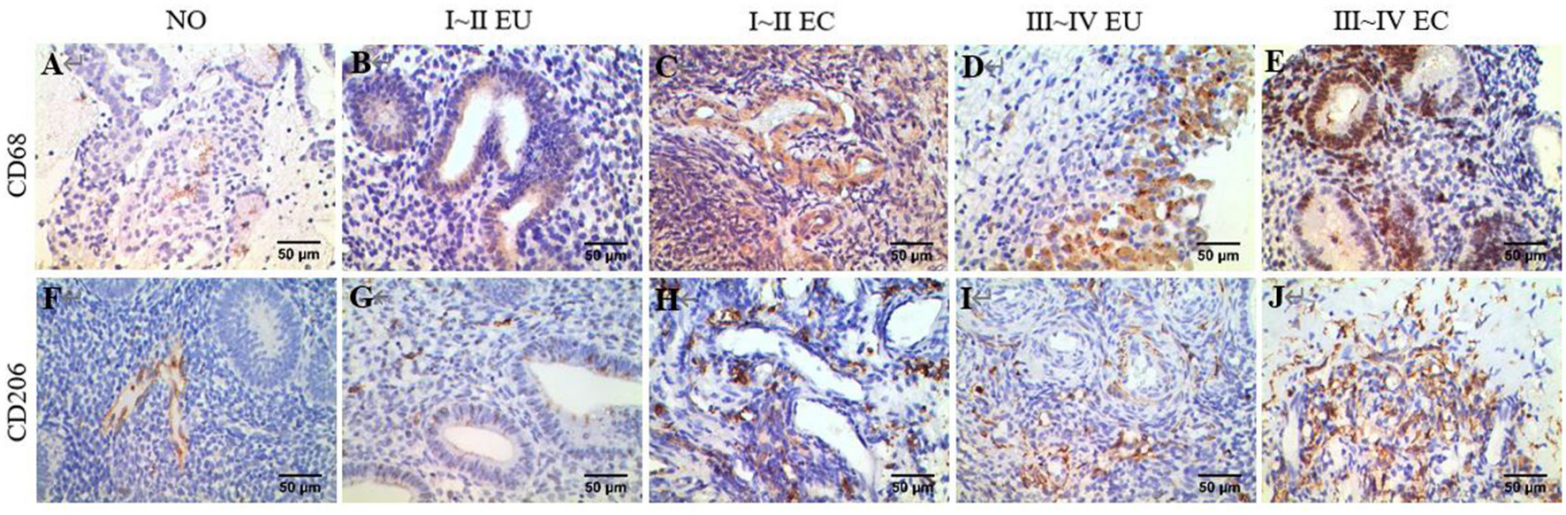
IHC of CD68 and CD206. Brown yellow staining indicates positive expression, and blue staining indicates nuclear staining. **(A)** Total macrophage CD68 staining in human NO tissue, ×400. **(B)** Total macrophage CD68 staining in human EU tissue of EMS stage I ~ II, ×400. **(C)** Total macrophage CD68 staining in human EC tissue of stage I ~ II EMS, ×400. **(D)** Total macrophage CD68 staining in human EU tissue of stage III ~ IV EMS, ×400. **(E)** Total macrophage CD68 staining in human EC tissue of stage III ~ IV EMS, ×400. **(F)** M2 macrophage CD206 staining in human NO tissue, ×400. **(G)** M2 macrophage CD206 staining in human EU tissue of stage I ~ II EMS, ×400. **(H)** M2 macrophage CD206 staining in human EC tissue of stage I ~ II EMS, ×400. **(I)** M2 macrophage CD68 staining in human EU tissue of stage III ~ IV EMS, ×400. **(J)** M2 macrophage CD206 staining in human EC tissue of stage III ~ IV EMS, ×400. All EU and EC tissues were from patients with ovarian endometriomas.

Examples of CD68 and CD206 macrophage staining in EC tissues, along with a quantification summary, are shown in Figure 7 and Table 6. Immunohistochemical results demonstrated that CD68 and CD206 were expressed in normal endometrium, eutopic endometrium and ectopic endometrium, exhibited cytoplasmic/membrane staining. Staining was distributed in the periphery and stroma of glands, and its positive expression showed as brown yellow and its nucleus as blue.

**Table 6:** Comparison of IHC results.

**Group**	**CD68**	**CD206**	**CD206/CD68**
NO	26.46 ± 10.76	16.00 ± 6.51	0.62 ± 0.16
I ~ II EU	30.31 ± 10.27^c^^e^	19.761 ± 7.90^c^^e^	0.68 ± 0.21^e^
I ~ II EC	43.71 ± 13.64^a^^b^	31.65 ± 11.62^a^^b^^e^	0.74 ± 0.16^a^
III ~ IV EU	36.15 ± 16.85^a^^e^	25.77 ± 14.18^a^^e^	0.71 ± 0.12^a^
III ~ IV EC	51.63 ± 26.49^a^^b^^d^	41.47 ± 24.14^a^^b^^c^^d^	0.79 ± 0.15^a^^b^
*F*	11.131	14.858	5.209
*P*	*P* < 0.001	*P* < 0.001	0.001

a *Compared to NO, p < 0.05*;

b *Compared to stage I ~ II EU, p < 0.05*;

c *Compared to stage I ~ II EC, p < 0.05*;

d *Compared to stage III ~ IV EU, p < 0.05*;

e *Compared to stage III ~ IV EC, p < 0.05*.

In agreement with the CIBERSORT results, macrophage density was higher in EC compared to eutopic and normal endometrial tissue. Meanwhile, CD206/CD68 was increased in EC, which confirmed M2 macrophage polarization in ectopic endometrium. Compared to stage I ~ II, CD68 and CD206 density and CD68/CD206 in endometrial tissue of stage III ~ IV EMS were increased.

## Discussion

In this study, we applied CIBERSORT to assess differential immune cell infiltration profiles in normal endometrial tissue, EC and EU of EMS. We observed considerable differences in immune cell composition among EC, EU, and NO. Additionally, different EMS r-AFS classifications were associated with distinct immune phenotypes. Macrophages were increased in EC, and M2 macrophage polarization was observed. In the ectopic endometrium of patients with stage III ~ IV EMS, the total number of macrophages and M2 macrophages was also increased, suggesting that M2 macrophages played an important role in EMS.

The recruitment and distribution of macrophages within the endometrial lesion have been demonstrated to facilitate and maintain EMS (Takebayashi et al., 2015; Scheerer et al., 2016; Wu et al., 2017). Berbic et al. found that the number of macrophages in ectopic lesions and peritoneal fluid from patients with EMS was significantly higher compared to eutopic endometrial tissue (Berbic et al., 2009). After coculture of macrophages and endometrial stromal cells (ESC) *in vitro*, the proliferation and invasion ability of ESC were enhanced, and the interaction between macrophages and nerve fibers enhanced EMS-related pain (Tran et al., 2009). Macrophages remove invasive pathogens and cell debris and express various cytokines, chemokines, and growth factors that mediate tissue repair, which is a key procedure of the formation of ectopic foci (Brancato and Albina, 2011; Ding et al., 2015).

Macrophages are mostly derived from progenitor cells in the bone marrow (Doulatov et al., 2010). Each macrophage subgroup in a specific tissue has a specific gene expression pattern and corresponding functional characteristics (Sprangers et al., 2016). At present, macrophages can be divided into M1 type and M2 type (Brown et al., 2012; Gordon et al., 2014). M1 macrophages can be activated by IFN-γ, TNF-α, or lipopolysaccharide, while M2 macrophages can be activated by IL-4, IL-10, or transforming growth factor-β (Franco and Fernández-Suárez, 2015). Some scholars have confirmed that expression levels of IL-10 and IL-4 in peritoneal fluid of patients with EMS are obviously increased, while expression levels of IFN-γ are markedly decreased (Khan et al., 2012). M1 macrophages can kill tumor cells and eliminate pathogens by inducing immune response or pro-inflammatory response. In contrast, M2 macrophages have anti-inflammatory properties, promote wound healing and fibrosis, repair tissues, participate in angiogenesis and promote tumor growth and infiltration (Hesketh et al., 2017). M2 macrophages produce various matrix metalloproteinases (MMP), such as MMP-2, MMP-7, and MMP-9 (Atri et al., 2018). MMP-9 enhances intercellular adhesion and promotes ectopic implantation and growth of endometrial cells by degrading extracellular matrix (Liu et al., 2015).

In this study, 22 types of immune cells were analyzed by CIBERSORT using transcriptome data from EMS patients in GEO databases. The distribution of total macrophages in ectopic endometrium was significantly increased. Compared to patients with r-AFS stage I~II, the proportion of M2 macrophages was increased in patients with stage III ~ IV disease, suggesting that the infiltration degree of M2 macrophages was related to the severity of EMS. IHC experiments showed that total macrophages were increased in ectopic endometrium, with M2 macrophages comprising the primary subtype. M2 macrophages in stage III ~ IV patients were increased compared to stage I~II, which was consistent with the CIBERSORT results. At present, many studies suggest that M2 macrophages are involved in the seeding and spreading of ectopic endometrium. Establishment of a BALB/c mouse EMS model showed that the degree of fibrosis was positively correlated with expression of M2 macrophages (Duan et al., 2018). After establishment of the mouse EMS model, depletion of macrophages affected the formation of blood vessels in the lesions and stopped the growth of ectopic lesions (Bacci et al., 2009).

Macrophage polarization involves many intracellular signal transduction molecules and complex signaling pathways, including the PI3K/AKt pathway, JAK/STAT pathway, Notch pathway and so on. IL-17A recruits peritoneal macrophages and promotes the polarization of macrophages into the M2 phenotype by acting on the EMS itself (Miller et al., 2020). Further studying the signaling pathways and related signaling molecules and intervening M2 macrophage polarization is vital for the prevention and treatment of EMS and can provide new ideas for hindering the occurrence and development of EMS.

In this study, bioinformatics analysis revealed that in addition to macrophages, NK cells, dendritic cells, plasma cells and resting CD4 memory T were also abnormally distributed in the ectopic endometrium of patients with EMS. Among them, the number of resting NK cells and activated NK cells were significantly decreased in ectopic endometrium. The abnormal number of NK cells and dysfunction of expression were present in ectopic lesions of EMS patients, and the number of activated NK cells was decreased, which might be related to the imbalance of activated receptor/inhibitory receptor ratio. These factors allow the endometrium to escape immune surveillance and immune clearance, leading to the occurrence and development of ectopic lesions. Increasing the killing ability of NK cells might be a potential treatment direction for EMS (Gómez-Torres et al., 2002; Matsuoka et al., 2005).

In summary, we demonstrate that deconvolution of whole tissue gene expression data by CIBERSORT provides refined information on the immune cell landscape of EC. We demonstrated that the presence of macrophages and M2 macrophage polarization might be relevant to EMS patient severity. Deviations of the EC immunoprofile from normal endometrial tissue may represent a valuable tool for identifying novel targets for immunotherapies and to individualize treatment strategies in patients with EMS.

## Data Availability Statement

The datasets presented in this study can be found in online repositories. The names of the repository/repositories and accession number(s) can be found in the article/Supplementary Material.

## Ethics Statement

The studies involving human participants were reviewed and approved by Clinical research and experimental animal ethics Committee, the first affiliated hospital of sun Yat-sen university. The patients/participants provided their written informed consent to participate in this study.

## Author Contributions

QZ carried out the study, analyzed, and interpreted the data. QZ and FY drafted the manuscript. JL, XC, and CZ collected and analyzed the data. SC participated in the design, original draft writing, and participated in the design and reviewed the manuscript. All authors read and approved the final manuscript.

## Conflict of Interest

The authors declare that the research was conducted in the absence of any commercial or financial relationships that could be construed as a potential conflict of interest.

## References

[B1] Ahn S. H. Monsanto S. P. Miller C. Singh S. S. Thomas R. Tayade C. (2015). Pathophysiology and immune dysfunction in endometriosis. Biomed. Res. Int. 2015:795976. 10.1155/2015/795976 26247027 PMC4515278

[B2] Ali H. R. Chlon L. Pharoah P. D. Markowetz F. Caldas C. (2016). Patterns of immune infiltration in breast cancer and their clinical implications: a gene-expression-based retrospective study. PLoS Med. 13:e1002194. 10.1371/journal.pmed.1002194 27959923 PMC5154505

[B3] Angelova M. Charoentong P. Hackl H. Fischer M. L. Snajder R. Krogsdam A. M.. (2015). Characterization of the immunophenotypes and antigenomes of colorectal cancers reveals distinct tumor escape mechanisms and novel targets for immunotherapy. Genome Biol. 16:64. 10.1186/s13059-015-0620-6 25853550 PMC4377852

[B4] Atri C. Guerfali F. Z. Laouini D. (2018). Role of human macrophage polarization in inflammation during infectious diseases. Int. J. Mol. Sci. 19:61801. 10.3390/ijms19061801 29921749 PMC6032107

[B5] Bacci M. Capobianco A. Monno A. Cottone L. Di Puppo F. Camisa B.. (2009). Macrophages are alternatively activated in patients with endometriosis and required for growth and vascularization of lesions in a mouse model of disease. Am. J. Pathol. 175, 547–556. 10.2353/ajpath.2009.081011 19574425 PMC2716955

[B6] Berbic M. Schulke L. Markham R. Tokushige N. Russell P. Fraser I. S. (2009). Macrophage expression in endometrium of women with and without endometriosis. Hum. Reprod. 24, 325–332. 10.1093/humrep/den393 19049988

[B7] Brancato S. K. Albina J. E. (2011). Wound macrophages as key regulators of repair: origin, phenotype, and function. Am. J. Pathol. 178, 19–25. 10.1016/j.ajpath.2010.08.003 21224038 PMC3069845

[B8] Brown B. N. Ratner B. D. Goodman S. B. Amar S. Badylak S. F. (2012). Macrophage polarization: an opportunity for improved outcomes in biomaterials and regenerative medicine. Biomaterials 33, 3792–3802. 10.1016/j.biomaterials.2012.02.034 22386919 PMC3727238

[B9] Chapron C. Marcellin L. Borghese B. Santulli P. (2019). Rethinking mechanisms, diagnosis and management of endometriosis. Nat. Rev. Endocrinol. 15, 666–682. 10.1038/s41574-019-0245-z 31488888

[B10] Ding D. Liu X. Duan J. Guo S. W. (2015). Platelets are an unindicted culprit in the development of endometriosis: clinical and experimental evidence. Hum. Reprod. 30, 812–832. 10.1093/humrep/dev025 25740881

[B11] Dmowski W. P. Steele R. W. Baker G. F. (1981). Deficient cellular immunity in endometriosis. Am. J. Obstet. Gynecol. 141, 377–383. 10.1016/0002-9378(81)90598-6 7282821

[B12] Doulatov S. Notta F. Eppert K. Nguyen L. T. Ohashi P. S. Dick J. E. (2010). Revised map of the human progenitor hierarchy shows the origin of macrophages and dendritic cells in early lymphoid development. Nat. Immunol. 11, 585–593. 10.1038/ni.1889 20543838

[B13] Duan J. Liu X. Wang H. Guo S. W. (2018). The M2a macrophage subset may be critically involved in the fibrogenesis of endometriosis in mice. Reprod. Biomed. Online 37, 254–268. 10.1016/j.rbmo.2018.05.017 30314882

[B14] Franco R. Fernández-Suárez D. (2015). Alternatively activated microglia and macrophages in the central nervous system. Prog. Neurobiol. 131, 65–86. 10.1016/j.pneurobio.2015.05.003 26067058

[B15] Gómez-Torres M. J. Acién P. Campos A. Velasco I. (2002). Embryotoxicity of peritoneal fluid in women with endometriosis. Its relation with cytokines and lymphocyte populations. Hum. Reprod. 17, 777–781. 10.1093/humrep/17.3.777 11870135

[B16] Gordon S. Plüddemann A. Martinez E. F. (2014). Macrophage heterogeneity in tissues: phenotypic diversity and functions. Immunol. Rev. 262, 36–55. 10.1111/imr.12223 25319326 PMC4231239

[B17] Hesketh M. Sahin K. B. West Z. E. Murray R. Z. (2017). Macrophage phenotypes regulate scar formation and chronic wound healing. Int. J. Mol. Sci. 18:71545. 10.3390/ijms18071545 28714933 PMC5536033

[B18] Khan K. N. Kitajima M. Yamaguchi N. Fujishita A. Nakashima M. Ishimaru T.. (2012). Role of prostaglandin E2 in bacterial growth in women with endometriosis. Hum. Reprod. 27, 3417–3424. 10.1093/humrep/des331 23001777

[B19] Laganà A. S. Garzon S. Götte M. Viganò P. Franchi M. Ghezzi F.. (2019). The pathogenesis of endometriosis: molecular and cell biology insights. Int. J. Mol. Sci. 20:225615. 10.3390/ijms20225615 31717614 PMC6888544

[B20] Liu H. Wang J. Wang H. Tang N. Li Y. Zhang Y.. (2015). Correlation between matrix metalloproteinase-9 and endometriosis. Int. J. Clin. Exp. Pathol. 8, 13399–13404. https://www.ncbi.nlm.nih.gov/pmc/articles/PMC4680492/pdf/ijcep0008-13399.pdf 26722547 PMC4680492

[B21] Matsuoka S. Maeda N. Izumiya C. Yamashita C. Nishimori Y. Fukaya T. (2005). Expression of inhibitory-motif killer immunoglobulin-like receptor, KIR2DL1, is increased in natural killer cells from women with pelvic endometriosis. Am. J. Reprod. Immunol. 53, 249–254. 10.1111/j.1600-0897.2005.00271.x 15833103

[B22] Miller J. E. Ahn S. H. Marks R. M. Monsanto S. P. Fazleabas A. T. Koti M.. (2020). IL-17A modulates peritoneal macrophage recruitment and m2 polarization in endometriosis. Front. Immunol. 11:108. 10.3389/fimmu.2020.00108 32117261 PMC7034338

[B23] Newman A. M. Liu C. L. Green M. R. Gentles A. J. Feng W. Xu Y.. (2015). Robust enumeration of cell subsets from tissue expression profiles. Nat. Methods 12, 453–457. 10.1038/nmeth.3337 25822800 PMC4739640

[B24] Rohr-Udilova N. Klinglmüller F. Schulte-Hermann R. Stift J. Herac M. Salzmann M.. (2018). Deviations of the immune cell landscape between healthy liver and hepatocellular carcinoma. Sci. Rep. 8:6220. 10.1038/s41598-018-24437-5 29670256 PMC5906687

[B25] Scheerer C. Bauer P. Chiantera V. Sehouli J. Kaufmann A. Mechsner S. (2016). Characterization of endometriosis-associated immune cell infiltrates (EMaICI). Arch. Gynecol. Obstet. 294, 657–664. 10.1007/s00404-016-4142-6 27358184

[B26] Simoens S. Dunselman G. Dirksen C. Hummelshoj L. Bokor A. Brandes I.. (2012). The burden of endometriosis: costs and quality of life of women with endometriosis and treated in referral centres. Hum. Reprod. 27, 1292–1299. 10.1093/humrep/des073 22422778

[B27] Sprangers S. de Vries T. J. Everts V. (2016). Monocyte heterogeneity: consequences for monocyte-derived immune cells. J. Immunol. Res. 2016:1475435. 10.1155/2016/1475435 27478854 PMC4958468

[B28] Symons L. K. Miller J. E. Kay V. R. Marks R. M. Liblik K. Koti M.. (2018). The immunopathophysiology of endometriosis. Trends Mol. Med. 24, 748–762. 10.1016/j.molmed.2018.07.004 30054239

[B29] Takebayashi A. Kimura F. Kishi Y. Ishida M. Takahashi A. Yamanaka A.. (2015). Subpopulations of macrophages within eutopic endometrium of endometriosis patients. Am. J. Reprod. Immunol. 73, 221–231. 10.1111/aji.12331 25345348

[B30] Tran L. V. Tokushige N. Berbic M. Markham R. Fraser I. S. (2009). Macrophages and nerve fibres in peritoneal endometriosis. Hum. Reprod. 24, 835–841. 10.1093/humrep/den483 19136478

[B31] Vitagliano A. Noventa M. Quaranta M. Gizzo S. (2016). Statins as targeted “magical pills” for the conservative treatment of endometriosis: may potential adverse effects on female fertility represent the “dark side of the same coin?” A systematic review of literature. Reprod. Sci. 23, 415–428. 10.1177/1933719115584446 25929256

[B32] Wang Y. Nicholes K. Shih I. M. (2020). The origin and pathogenesis of endometriosis. Annu. Rev. Pathol. 15, 71–95. 10.1146/annurev-pathmechdis-012419-032654 31479615 PMC7980953

[B33] Wu J. Xie H. Yao S. Liang Y. (2017). Macrophage and nerve interaction in endometriosis. J. Neuroinflammation 14:53. 10.1186/s12974-017-0828-3 28288663 PMC5351283

[B34] Zeng C. Xu J. N. Zhou Y. Zhou Y. F. Zhu S. N. Xue Q. (2014). Reproductive performance after surgery for endometriosis: predictive value of the revised American Fertility Society classification and the endometriosis fertility index. Gynecol. Obstet. Invest. 77, 180–185. 10.1159/000358390 24603632

