# Supplementary figures and images for: Patterns of Immune Infiltration in Endometriosis and Their Relationship to r-AFS Stages
Source: Front Genet. 2021 Jun 18;12:631715. doi: 10.3389/fgene.2021.631715 (PMC8249861; doi:10.3389/fgene.2021.631715)

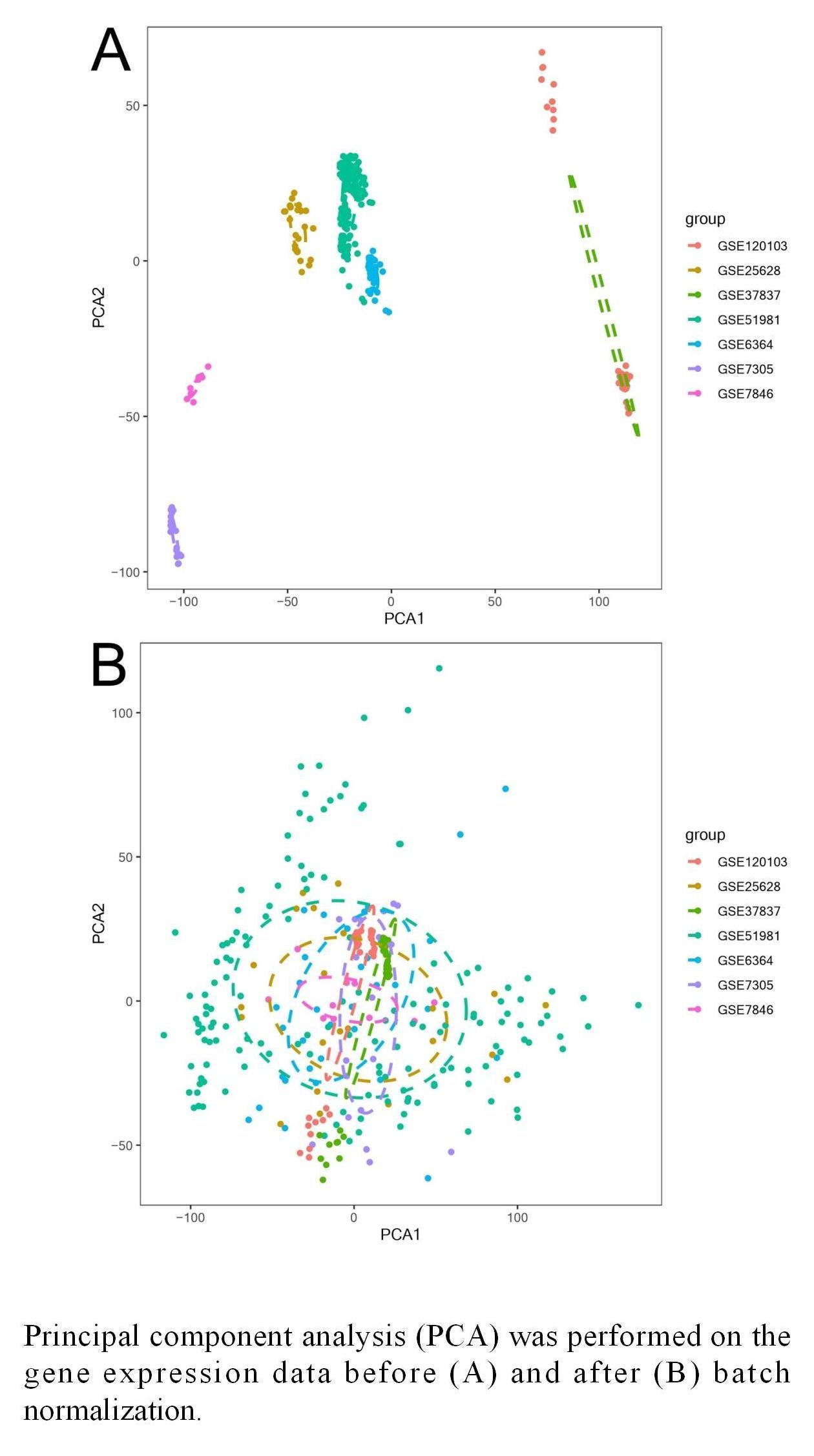

Supplement: Supplementary file 4 [file Image_1.JPEG]

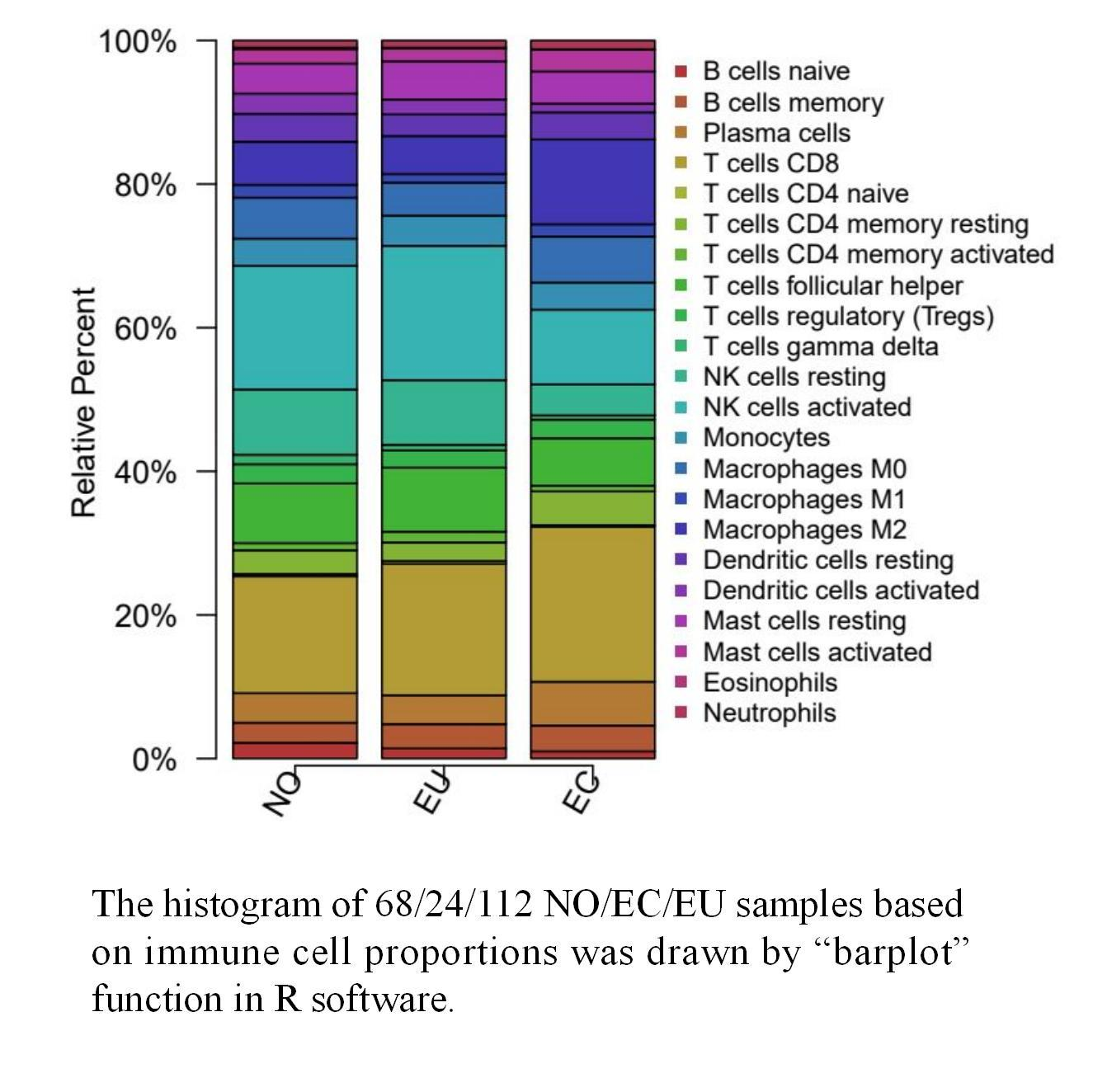

Supplement: Supplementary file 5 [file Image_2.JPEG]
